# The Impact of Service Dogs on Engagement in Occupation among Females with Mobility Impairments: A Qualitative Descriptive Study

**DOI:** 10.3390/ijerph14060649

**Published:** 2017-06-16

**Authors:** Ellen Herlache-Pretzer, Melissa Y. Winkle, Rachel Csatari, Alyssa Kolanowski, Amy Londry, Rachel Dawson

**Affiliations:** 1 Department of Occupational Therapy, Saginaw Valley State University, Saginaw, MI 48663, USA; amkolano@svsu.edu; 2 Dogwood Therapy Services, Albuquerque, NM 87114, USA; melissa@dogwoodtherapy.com; 3 Medilodge of Capital Area Lansing, Genesis Rehab, Lansing, MI 48910, USA; recsatari@gmail.com; 4 Encore! Rehabilitation, Farmington Hills, MI 48334, USA; allondry@svsu.edu; 5 Munson Grayling Rehabilitation Services, Grayling, MI 49738, USA; rrrombal@svsu.edu

**Keywords:** activities of daily living, assistive technology, mobility dogs, occupational therapy, service dogs

## Abstract

It is becoming more common for people with disabilities to procure service dogs as a form of assistive technology (AT). However, there is little qualitative research examining the impact of service dogs on engagement in valued daily activities (occupations) among persons with mobility impairments. This study used a qualitative descriptive methodology to learn about the experiences of four female service dog owners with mobility impairments, with a focus on the impact of service dog use on the performance of daily occupations and participation in social activities, and their experiences utilizing a service dog as a form of AT. Data analysis indicated that each participant’s service dog made a significant impact on their everyday lives and their ability to independently perform everyday activities; however, there are also unique challenges associated with service dog ownership that must be considered when evaluating benefits of service dog partnership. Overall, the positive outcomes reported by participants indicate that service dogs can be considered a beneficial, adaptable form of AT for some persons with mobility impairments.

## 1. Introduction

Occupations are defined as valued daily activities, performed with a specific purpose and meaning, that are impacted by one’s culture and factors internal and external to the person [1]. It is common for individuals to overlook the significance of participation in everyday occupations until the ability to do these activities is lost or altered. Individuals who have faced any type of challenge in their lives, including physical, may not be able to independently engage in desired occupations [2].

When individuals have deficits in completing day-to-day occupations, assistive technology (AT) is a tool that may be utilized to aid individuals in engagement [3]. According to the Technology-Related Assistance for Individuals with Disabilities Act of 1988 [4], AT is “any item, piece of equipment, or product system, whether acquired commercially off the shelf, modified, or customized, that is used to increase, maintain, or improve functional capabilities of individuals with disabilities. AT service is directly assisting an individual with a disability in the selection, acquisition, or use of an assistive technology device.” The use of AT devices and services can help individuals to regain independence and function in various aspects of life [3,5,6].

Service dogs perform a variety of tasks that can maintain or improve functional capabilities of the individuals with whom they work. Service dogs can be trained to help individuals with mobility impairments engage in occupations such as assisting to propel wheelchairs, opening doors, retrieving dropped items, providing balance, and conserving energy. Camp [7] reports that service dogs are used to enhance independence in occupational performance areas and to contribute to improvements in psychosocial functioning; “given these benefits, service dogs could be used as a form of assistive technology under the Technology Act in that they also are ‘used to increase, maintain, or improve functional capabilities of individuals with disabilities’” (p. 509). Similarly, Shintani et al. [8] found positive effects of service dogs on various aspects of health-related quality of life, as measured by the Medical Outcomes Study 36 Item Short-Form Health Survey Version 2.0 (SF-36v2). The researchers concluded that they “…hope that this research will encourage more therapists to acknowledge service dogs as a new assistive technology, and to consider the recommendation of service dogs, under the right circumstances, for the people with physical disabilities they work with” (p. 113). It is clear that service dogs can assist persons with mobility impairments to engage more fully in valued daily occupations [8,9,10,11,12,13].

Prior research has verified the positive impact of service dog ownership on various aspects of health, quality of life, and social participation. For example, a study by Hall, MacMichael, Turner, and Mills [14] found that, when compared to persons on a wait-list for a service dog, persons partnered with a service dog had significantly higher scores on an assessment of quality of life. In particular, the researchers reported that those with service dogs had higher scores on areas related to socialization and engagement in educational, volunteer, and work activities. Similarly, the results of a randomized controlled trial by Allen and Blascovich [15] concluded that among persons with severe disabilities affecting ambulation, service dog partnership resulted in improved psychological, social, and educational/vocational functioning, as well as a decrease in the number of hours of paid/unpaid assistance required. Furthermore, the results of research by Lane, McNicholas, and Collis [16] suggested that service dog partnership is associated with increased social interaction and improved self-perceptions of health. A longitudinal study by Guest, Collis, and McNicholas [17], focused specifically on persons who were deaf or hard-of-hearing, found that partnership with a hearing dog had positive impacts on psychological well-being (anxiety, depression, and tension), as well as social interaction.

While prior research has demonstrated the positive impacts of service dogs on social interaction, psychological well-being, and self-perceived health, there is a limited body of qualitative research specifically examining how service dogs impact engagement in occupation among individuals with mobility impairments. Studies conducted by Camp [7] and Fairman and Huebner [13] provided preliminary research supporting the idea that service dogs can assist with performance of occupations. However, Camp indicated that there was a need for further research regarding how assistance dogs compare to an individual’s prior form or forms of AT. In a literature review conducted by Winkle, Crowe, and Hendrix [11], it was concluded that additional qualitative studies should be conducted to examine the meaningfulness of service dog use, and provide comparison of previous AT with current service dog use. Furthermore, as stated by Shintani et al. [8], additional research is needed to assist rehabilitation professionals in understanding the impacts of service dogs on the daily performance of those with disabilities, and grasping the potential role of rehabilitation professionals in working with persons who may benefit from service dog partnership.

The purpose of this qualitative descriptive study was to learn how service dogs impact engagement in occupation among persons with mobility impairments that affect gait or balance, as well as their experiences utilizing a service dog as a form of AT. This study built upon previous research by exploring, from the owners’ perspectives, how their service dogs influenced their engagement in occupations inside the home and throughout the community, and how their service dog compared to AT used in the past.

## 2. Materials and Methods

This study utilized a qualitative descriptive research design involving interviews with four participants. This approach allowed the researchers to learn about each participant’s engagement in occupation while partnered with a service dog, and how their experiences with their service dog differed from their experiences with various types of AT. All subjects gave their informed consent for inclusion prior to participation in the study. The study was conducted in accordance with the Declaration of Helsinki, and the protocol was approved by the Saginaw Valley State University Institutional Review Board (Project 290771-2).

Participants were obtained through convenience sampling and snowball sampling. Recruitment flyers containing information about the research study were distributed to clients of one organization that trains service dogs. A total of four persons who met the inclusion criteria volunteered to participate in the study. The population for this study consisted of English-speaking adults over 18 years of age with a mobility impairment. Participants were required to have been partnered for at least one year with a service dog trained by the organization (which is accredited through Assistance Dogs International (ADI)). The service dog had to assist them with completion of activities of daily living (ADLs, such as functional mobility and retrieving items); and instrumental activities of daily living (IADLs, such as caring for another living being, community mobility, work, play, and/or leisure).

Data was collected through in-depth, semi-structured phone interviews which lasted for approximately one hour. A structured interview schedule, consisting of three core questions (each with multiple sub-questions) was used to guide each interview. Questions were developed based upon the main areas of focus of the research study, which included the impact of service dog partnership on individuals’ engagement in occupation; comfort level going into the community with their service dog; and their feelings regarding working with a service dog as a form of AT. All participants were allowed to speak for as long as they felt necessary to fully respond to the presented questions.

All interviews were transcribed for data analysis, which was completed through a process of open, axial, and selective coding, as described by Patten [18]. Open coding was used to identify common ideas expressed by all participants in interviews; these shared ideas were given a descriptive label (“code”). Axial coding was used to condense the number of categories identified during the open coding process, by combining categories sharing similar ideas. Finally, selective coding was used to further condense the number of categories identified during the axial coding process, and identify core overarching ideas identified by participants. Triangulation of researchers, member checking, and peer examination, as described by Krefting [19], were utilized during the research process to enhance trustworthiness of reported results.

## 3. Results

A total of four females (see Table 1) volunteered to be interviewed for this qualitative descriptive study.

Following analysis and coding of the data, four broad themes appeared. All participants expressed that their service dogs had made significant impacts on their daily lives and their abilities to independently perform everyday occupations, and had characteristics that made them unique from prior forms of AT used in the past.

### 3.1. Value of Service Dogs as a Form of AT

The first broad theme expressed by the participants was that service dogs provide valuable assistance in the completion of everyday activities within the home and community. The support that service dogs provide, as a form of AT, allows handlers to engage in everyday occupations more independently. The participants explained that because of the assistance they receive from their service dog, they can perform tasks more safely, conserve energy, and require less help from other people while performing daily occupations including bathing and showering, dressing, functional mobility, personal hygiene and grooming, and toilet hygiene. P2 described how her service dog is extremely helpful in assisting with her daily task of dressing:

“*… When I get ready for bed at night, all I have to do is kick off my sneakers and [my dog] picks them up and puts them away, she knows where they go. And if, like, the next morning I’m someplace else in the house, and ask her for my sneakers, she will go get them and bring them to me.*”

All participants stated that one of the biggest assets of owning a service dog is the dogs’ ability to assist with functional mobility, including transferring, reaching to the floor, ascending and descending stairs, and carrying items. Additionally, the participants described how their service dogs help them in more complex daily activities such as doing the laundry, shopping, meal preparation, traveling, and performing work-related tasks. For all participants, the assistance provided by their service dogs allowed them to continue to engage in valued habits and routines.

All participants stated that their service dogs impact their safety both at home and in the community, which in turn impacts the abilities of themselves and/or loved ones to engage in various activities in the community. For example, when talking about her service dog, P1 stated that:

“*I couldn’t tell you how many times [my dog] has kept me from falling. If I lose my balance, she plants her feet like a rock and doesn’t move… so I feel very safe. My husband, that was one of the things he remarked from the get-go was that it made him feel so much better with me being home alone… [because] she was there [and] because she takes care of me.*”

P4 commented how having the constant support of her dog has enabled her to go outside alone:

“*Before I got [my dog], I never went outside by myself unless someone was coming to pick me up. Once I got her, I had to go out alone because she needed to play. I realized how good it felt to go outside on my own. I actually started going outside on nice days to just sit and read for my classes. I also got out of my wheelchair and sat in the grass because I knew she could help me back into my wheelchair when I was ready to go.*”

Because of the assistance and support provided by their service dogs during completion of daily occupations, the participants reported an increase in their energy level and safety, and they ultimately came to rely less on others for everyday needs.

### 3.2. Impact of Service Dog Partnership on Community Integration

The second theme that emerged focused on how participants’ service dogs impacted their experiences in the community. Responses were broken down into two subthemes: positive situations in public, and challenges which led to the education of others.

#### 3.2.1. Positive Situations in Public

All participants noted that their service dog made them feel like they could go out into public more. While visiting public places, they felt a sense of security because the service dog was solely focused on the handler’s well-being. Participants discussed that with their service dog by their side they felt that they could participate in more social situations as well. Many common anxieties about going into public that were experienced by the participants diminished when they had their service dog by their side. P3 described how her partnership with her dog gave her the confidence to go into the community without relying on other people’s assistance:

“*[My dog] helps me get things done without having to ask people all the time. I don’t have to hold my husband’s hand all the time when I can’t walk. I can do things by myself. For the first time ever, last year I was able to go swimsuit shopping by myself in the mall because I had [my dog] as a counter balance and as an alert dog so I knew I was completely safe. I knew I could do it all by myself.*”

All participants reported feeling more confident and competent going into community settings with their service dogs by their side to retrieve items, assist with transferring, opening and closing doors, and help with any other necessary tasks.

#### 3.2.2. Challenging Situations and Education of Others in Public

While going out in public with a service dog it is common for others to want to pet, stare at, and ask questions about it. All participants reported that they often must educate others that the service dog is working and should not be distracted from his or her work. Participants alluded that it is common for them to be stopped while they are out; as P1 describes:

“*… for the most part its usually positive, it can be a little tiresome repeating a million questions… what’s that thing on her back and… what does she do for you and why do you have that thing on her back, that looks heavy...*”

Unfortunately, not all community business employees are as receptive of service dogs as others or understand what it is doing for its handler. As described by P1, service dog teams may face challenges even in businesses they frequently visit:

“*… well one morning I went in and [my dog] went to go do the ‘go pay’ and it was a young kid… all of a sudden it was ‘ah I’m not touching that money that dog doesn’t even belong in this place’.*”

This situation led to the education of the employee, the business, as well as other residents and other businesses in the community.

The participants expressed that some social settings present challenges. Often when people do not know how to respond appropriately to an individual with a service dog, misinterpretations can occur. However, the participants discussed how they have learned to provide education to people who may not understand the role of a service dog.

### 3.3. Comparison of Service Dogs versus Other Forms of AT

#### 3.3.1. Adaptability of Service Dogs

The third theme focused on benefits and drawbacks associated with obtaining and maintaining service dogs versus other forms of AT. Participants reported that each of their service dogs had learned to adapt to fit specific needs in different contexts to help maximize their handlers’ independence, which was a significant benefit associated with service dog partnership. As P1 described:

“*… If its late at night [and] I need medication or just a snack or something… I will text down [stairs] and say ‘hey… if I send [my dog] down will you get me a baggy of crackers or can you put two Tylenol in a Ziploc baggy or whatever and send it up for me?’… I send her, I say ‘go take it’ and then… she brings whatever I asked for up in a baggy.*”

All participants expressed how they have developed ways of tailoring their service dog’s skills to fit their individual needs. They valued the ability of their service dogs to adapt and change over time and felt that this was a valuable characteristic of their service dogs that they hadn’t encountered with other forms of AT used in the past.

#### 3.3.2. Extra Effort and Expenses

There are also potential drawbacks associated with the use of a service dog as a form of AT. For example, there are expenses associated with ownership and the upkeep of a service dog, which are not present with other forms of AT. The organization from which the participants in this study obtained their service dogs utilizes a sliding fee scale; clients are asked to pay anywhere from $1000 to $5000 towards the initial cost of their dog. Participants noted that there are also costs and effort associated with initial training, including travel to the training site/organization and community training outings. Participants also highlighted the multiple expenses associated with long-term service dog ownership, including vet care, food, grooming supplies, and toys. While the participants noted that their service dogs require extra work and expenses, they claimed that the benefits and support that they provide outweigh the additional work.

#### 3.3.3. Fluctuations in Performance of Service Dogs

Service dogs are living creatures, and demonstrate fluctuations in daily performance that are not encountered with other forms of AT. All participants commented that they have learned to be prepared for these instances. P4 described how she handles these situations:

“*If I tell [my dog] to take something and she decides, for some reason that she doesn’t want to take it, I can’t take no for an answer… so I have to persist until she cooperates… so you know, in that way sometimes, what I think is a two second project, turns into a five minute ordeal [laughing]. And it’s like, oh my goodness, I don’t know if I had time for that.*”

Since the handlers can never guarantee how a dog will react to all situations, they must learn to plan for them. P1 is familiar with these fluctuations in her dog’s performance, and has modified some situations in which she asks her dog to complete task work:

“*Once in a blue moon, yea they get their ‘attitude’… I don’t give her anything glass because occasionally she gets an attitude where she’ll just look at me and drop the bags and I don’t give her eggs [laughing] at the grocery store for that same reason.*”

The participants all stated that they had to learn to understand that their service dog is just that—a dog—and that they must be flexible and prepared for variations in the dog’s performance, depending upon the day and situation.

### 3.4. Value of Relationship with Service Dog

The final theme that emerged focused on the value of the relationship formed with their service dogs, and how the presence of this relationship made service dog partnership preferable to the use of other forms of AT in many situations. Due to the constant companionship and interaction, strong bonds form between the handlers and their service dogs. P3’s description of a situation in which she had to utilize a scooter highlighted the importance of the relationship between her and her service dog:

“*[My dog is]… just great at helping in general… I did use a scooter once, right after a seizure when I was in a [grocery store]… I tell you if I ever had to use something again I still think I’d just use the dog because they have an emotional substance to them that you just can’t get from a walker or a scooter or a wheelchair... They just do so much more for you and they give much more to you when you’re in pain; they feel it, and they try to help you so much and give you so much back that it is just so worth it.*”

All participants reported that they have developed a strong relationship with their service dog because of their constant interaction. They noted that their dogs seem to understand them and their capabilities, and can respond as needed to provide appropriate assistance and support. For example, P2 expressed how well her service dog understands her physical tolerance level and knows when she needs to refrain from an activity: “She knows me better then I know myself, she’ll tell me when I’m done… fatigued… [if] I try and get up and take a couple steps or something, she’ll stand in front of me, not beside me.” The participants disclosed how their service dogs have impacted their lives by providing a constant sense of security and support. They placed a high value on the relationship that they developed with their service dogs, and felt that the service dogs modified task performance based upon their (the participants’) current condition. Overall, participants felt that the handler-dog relationship was the key factor that made their service dog superior to other forms of AT used in the past.

## 4. Discussion

The results of participant interviews suggested that service dogs have had a positive impact on their handler’s lives, and have allowed them to be more independent in daily occupations. Not only do the participants’ service dogs aid in the performance and completion of ADLs and IADLs, but they have also been a constant source of support, and increased their handlers’ participation in a variety of social contexts.

Previous research has indicated that because assistance and service dogs possess the ability to assist persons with disabilities by improving their independence and participation in occupations, they can be considered an alternative form of AT [7,8,12,20]. The participants in the present study reported that they continued to use some forms of AT (e.g., wheelchair, adapted van) they had utilized before obtaining their service dog. Despite this, the participants identified that the service dogs, functioning as a form of AT, increased independence and participation in occupations for individuals with mobility impairments more than many other forms of AT available to them. Additionally, they recognized the value and importance of the relationship they formed with their service dogs in terms of helping them to feel secure in completing daily activities at home and in the community. For all participants, this relationship is what ultimately differentiated their service dog from other forms of AT utilized in the past, and what made service dog partnership superior to the use of other forms of AT in many situations.

Interestingly, the participants in the present study identified the importance of personalization of service dogs to fit each handler’s specific needs. This concept was not originally addressed in the research questions, but emerged during the participant interviews. All participants reported using their service dog in conjunction with AT devices that they were already using. However, they also expressed how they established specific, unique cues or gestures with their service dog to help their dog to best meet their own needs. Therefore, service dogs have the potential to offer evolving, customized assistance that individuals may not be able to obtain from other forms of AT.

The results of this study indicated that there are significant benefits associated with the use of service dogs; however, there are factors unique to service dogs (versus other forms of AT) that must be addressed with persons considering this form of AT. In the present study, participants discussed the extra time, cost, and energy involved in grooming, feeding, exercise, and caring for their service dogs. They also noted that service dogs, as living creatures, demonstrate some fluctuations in performance that may not be seen in other forms of AT; they had to be prepared (and patient) to work through these situations. Nonetheless, all the participants expressed how the benefits of owning a service dog surpassed the extra costs, time, and energy required. These are all factors that likely need to be considered when exploring the use of a service dog as a form of AT.

The present study found that service dogs impact every aspect of their handlers’ lives including participation in daily routines and occupations within the home and community. Occupational therapy practitioners concentrate on client performance in areas of occupation, including daily routines and occupations. It might be beneficial for occupational therapy practitioners to work more closely with service dog training organizations. Occupational therapists are trained to provide client-centered assessments and interventions to best meet each client’s individual needs. Similar to findings reported by Shintani et al. [8] and Fairman and Huebner [13], the results of the present study suggest that therapists could work in collaboration with service dog training organizations to assist with the client assessment and education/training process.

Several limitations were present within this study. A main limitation was the small sample size of only four Caucasian female participants. Additional information about participants (including educational and/or occupational status, living arrangements, etc.) was not gathered as part of the interviews, which may impact the generalizability of findings. All the dogs were trained through the same service dog training organization, leading to a lack of diversity of training and placement experiences. Additionally, the individuals who participated in this study described positive experiences with their service dogs. However, participants who may have had negative experiences might not have been motivated to participate in the study. Consequently, the results of the study are not necessarily generalizable to the greater population.

## 5. Conclusions

The purpose of this qualitative descriptive study was to learn how service dogs impact engagement in occupation among individuals with mobility impairments, and learn about experiences utilizing a service dog as a form of AT. After completing interviews with four female participants and analyzing the data, it was found that the participants’ service dogs substantially impact their everyday lives. Service dog partnerships influence the participants’ abilities to independently perform everyday occupations. Additionally, the relationship the participants formed with their service dogs provided them with a sense of increased security and support, thus encouraging them to be more active at home and in the community. The positive outcomes reported by participants indicated that service dogs could be recommended more as a form of AT for persons with mobility impairments.

The use of service dogs as a form of AT by people with disabilities is an emerging area [11]. Additional research is suggested to examine “best practice” recommendations regarding the process of obtaining and utilizing a service dog. This suggests the need for more rigorous studies with a larger sample size, variance in gender, and a wider geographical range. 

## Figures and Tables

**Table 1:** Participant demographic data.

Participant	Age	Disability	Age of Dog	Years Owned
P1	50	Multiple sclerosis	8	5
			deceased	4
P2	61	Multiple sclerosis	9	7
P3	30	Progressive epilepsy	4	2
P4	27	Undiagnosed muscular disorder	3.5	1
